# Continuous endometrial volumetric analysis for endometrial receptivity assessment on assisted reproductive technology cycles

**DOI:** 10.1186/s12884-020-03372-2

**Published:** 2020-11-03

**Authors:** Renato Silva Martins, António Helio Oliani, Denise Vaz Oliani, José Martinez de Oliveira

**Affiliations:** 1 Centro Hospitalar Universitário Cova da Beira EPE, Quinta do Alvito, 6200 503 Covilha, Portugal; 2 Centro Investigação Ciências da Saúde – Faculdade Ciências da Saúde, Universidade da Beira Interior, Alameda Infante D, Henrique, 6200 506 Covilha, Portugal

**Keywords:** Endometrial receptivity, Assisted reproductive technology, Endometrial volume, Adjusted endometrial volume, Embryo implantation

## Abstract

**Background:**

Human implantation is a complex process requiring synchrony between a healthy embryo and a functionally competent or receptive endometrium. In order to assess endometrial receptivity in Assisted Reproductive Technology (ART) cycles serial evaluation of endometrial volumetric analysis may have a predictive value on a positive outcome.

**Methods:**

Serial 3D transvaginal ultrasound performed in women on ART cycle to evaluate embryo implantation predictors. Prospective case control study of 169 subjects were assessed. Endometrial pattern, thickness, volume and adjusted endometrial volume (ratio between endometrial volume and uterine volume) was performed to all subjects on a continuous process from baseline, during controlled ovarian stimulation, trigger day with human chorionic gonadotropin hormone (hCG) and at embryo transfer day.

**Results:**

Demographics and ART procedures and scores, was similar between the two groups. Endometrial morphology also showed no difference between the two groups. Endometrial volume and adjusted endometrial volume was significantly higher in the positive group as soon as day 6 of ovarian controlled stimulation.

**Conclusions:**

Serial 3D endometrial volume and adjusted endometrial volumes provides a predicting clinical tool enhancing elective embryo transfers in fresh ART cycle. Thus providing a non-invasive continuous technique for endometrial receptivity assessment that reflects endometrial changes during ART procedures.

## Background

Successful assisted reproductive technology cycles outcome depends on the intricate interplay between embryo quality and endometrial receptivity. Endometrium is a dynamic tissue that grows, differentiates and suffers regression throughout the menstrual cycle in response to hormonal regulation to prepare the uterus for embryo implantation [1]. Endometrium is a highly dynamic tissue undergoing physiological changes in response to ovarian steroid hormones. It has been proven that the supraphysiological hormonal levels as in the ART cycle has a harmful effect on endometrial receptivity. Endometrial characteristics compatible with a successful pregnancy have proven to be difficult to be properly assessed. Adequate endometrial development seems to be important for implantation given that previous studies have shown an association between abnormal glandular or vascular development and defective placentation disorders. The window of implantation (WOI) is defined as a short period of time while the endometrium is receptive to the embryo [2, 3].

Diagnosis of endometrial receptivity (ER) has posed a challenge and so far, most available tests have been subjective and lack accuracy and a predictive value [4].

The use of transcriptomic signature of the WOI by microarray technology is possible, however it demands an endometrial biopsy [5]. This requires an invasive procedure and it has an associated cost. Also, in women with irregular cycles it may not prove to be cost-efficient [6].

Ultrasound can asses changes in the endometrium during stimulated cycles. It also has minimal inter-observer and intra-observer variability. Monitoring of both the endometrial and ovarian response to ovarian stimulation on ART cycles with transvaginal ultrasound has become an important predictor of the success of ART. Published studies have conflicting results on this subject, but the common feature in all, is the lack of continuity on endometrial assessment [7]. The use of high-resolution transvaginal probes makes it possible to follow endometrium changes throughout the cycle [8]. From a clinical point of view some objective parameters must be obtained in order to ascertain the likelihood of an ongoing pregnancy in ART cycles, preferably in a non-invasive and cost-efficient way [9]. Some published work has recently proven a pattern of hemodynamic changes in utero-ovarian arteries during ART cycles with predictive value on endometrial receptivity [10]. Hou et al. have also confirmed the possibility of non-invasive prediction of success in ART cycles, with serial assessments of the echogenicity pattern transformation, after human recombinant gonadrotropin hormone [11]. The aim of this prospective study is to further evaluate the capability of serial and continuous evaluation of biophysical markers as a non-invasive procedure to determine endometrial receptivity [12, 13].

## Methods

**Fig. 1:**
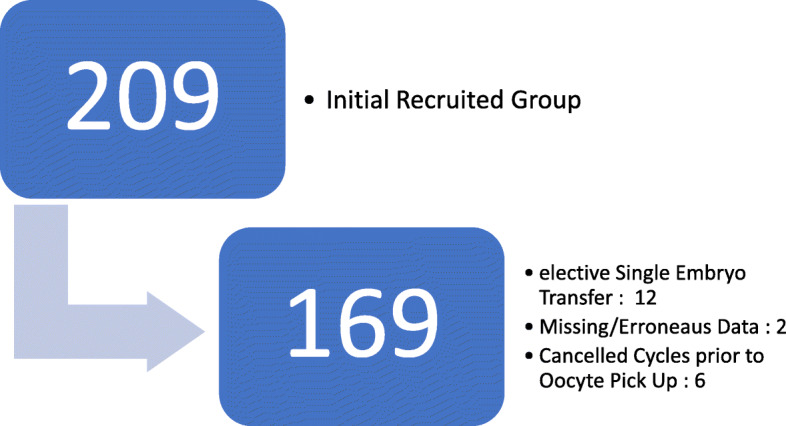
Study Population and Exclusion Criteria

Prospective cohort study of 169 women in ART cycles. All infertile couples submitted to ART treatment at our institution were included from January 2017 to December 2018 (2 Year period). Canceled treatments prior to oocyte pickup; cycles with missing or erroneous data; and cycles with elective single embryo transfer were excluded. (Fig. 1.)

The primary data source for this study was the local databases routinely used in the participating centre in ongoing treatments. The data output was anonymized in the extraction for statistical treatment purposes. All data collected and written informed consent was obtained according to the Ethics Committee of our Institution.

Subjects with double viable good grade embryo transfer on day 3 of embryo development were included. All subjects have been in a short protocol regimen with antagonist for ovarian controlled stimulation using gonadotropins. All subjects were submitted to recombinant human chorionic gonadotropin hormone (rhCG) for induction of ovulation 36 h prior to oocyte pick up.

Ultasonographic protocol for serial ultrasound analysis (endometrial morphology, endometrial thickness, endometrial volume and uterine volume) was performed.

During ovarian controlled stimulation serial ultrasound exams were performed, using 3D transvaginal probe.

Ultrasonographic markers were obtained in all evaluations (Basal moment – day 2 or 3 of women menstrual cycle and prior to onset of ovarian controlled stimulation; at day 6, day 8 and day 10 after initiating ovarian controlled stimulation; at Trigger day with recombinant human gonadochorionic hormone; and at embryo transfer day).

Endometrial morphology was based on the two grade system by *Sher et al.* [14]: non-multilayered homogeneous hyperechogenic or iso-echogenic endometrium compared with the myometrium and multilayered triple-line pattern, ‘halo pattern’ with an outer peripheral layer of denser echogenicity and a central sonolucent area.

Endometrial thinckness was obtained in milimeters (mm) on the long axis or sagittal plane, with the entirely of the endometrial lining through and endocervical canal in view. The measurement was taken of the thickest echogenic area from one basal endometrial interface across the endometrial canal to the other basal surface.

Endometrial volume calculation by 3D ultrasound presented as voxels and geometric information of surfaces in a 3D dataset. The results obtained are then converted to mililitres. Adjusted Endometrial volume was also obtained as a ratio between endometrial volume calculated on 3D analysis and uterine volume based on 3D volumetric acquisitions which then generated an estimated uterine volume (also in mililiters). Adjusted endometrial volume deflects the potential difference in uterine volume from each single individual.

At day 12 after successful embryo transfer, human gonadochorionic sub-unit B serum levels were obtained, and groups were set: positive results (for values over 5 International Units - IU) and negative results (for values under 5 IU). For the aim of our study the positive cases were afterwards assessed and classified with positive clinical pregnancy by the evidence of at least one viable foetus by ultrasound performed 2 weeks after the positive biochemical result.

All data collected was analysed between these two set groups and compared.

Data was analysed in Excel 2019 (Microsoft Corp, Redmond, WA) and IBM SPSS statistics v25 (IBM Corp. Armonk, NY). Continuous variables were analysed with Levene’s test (equality of variances) and visual assessment of the histogram (normality).

For analysis of parametric continuous variables, a t-student test for independent samples was used. Chi-square and Fisher’s exact tests were used to analyse associations between categorical variables. Endometrial thickness, endometrial volume and adjusted endometrial volume were analysed using analysis of variance for repeated measurement data.

Value of *p* < .05 was considered statistically significant.

The authors do not report any conflict of interest.

The study protocol has been approved by the Ethics Committee of our Institution (CHCB 22/2017), in accordance with the relevant guidelines and regulations. This study has been conducted in accordance with legal and regulatory requirements, as well as follow generally accepted research practices described in International Conference Harmonisation (ICH) guidelines, Good Clinical Practices (GCP) and the Declaration of Helsinki.

## Results

Clear morphology and volume of the endometrium was obtained in all 169 cycles using 3D transvaginal ultrasound in continuous serial observations. Demographics characteristics and ART parameters are shown in Table 1.

**Table 1:** Demographics and ART parameters between two Groups

	Negative Group *N* = 123 (72.8%)	Positive Group *N* = 46 (27.2%)	t-Test *p* value
Female Age (in years)	34.94 ± 4.03 (19–39)	34.28 ± 3.35 (25–39)	0.290
Male Age (in years)	36.14 ± 4.76 (22–46)	37.19 ± 5.91 (29–62)	0.832
Time of Infertility (in months)	54.46 ± 33.82 (12–204)	60.22 ± 38.49 (14–192)	0.375
Type of Infertility:			
• Primary	95/123 (77.2%)	38/46 (82.6%)	0.297
• Secondary	28/123 (22.8%)	8/46 (17.4%)	
Antimullerian hormone (pg/mL)	2.45 ± 2.45 (0.09–16.65)	2.62 ± 2.46 (0.04–13.56)	0.679
Antral follicle count	8.43 ± 5.07 (2–40)	8.63 ± 3.74 (2–20)	0.801
Total dose of gonadotropins (in International Units)	2500.81 ± 812.19 (300–4500)	2508.15 ± 757.91 (450–4500)	0.956
Progesterone levels at Trigger day (ng/mL)	0.88 ± 0.44 (0.01–2.20)	0.78 ± 0.47 (0.01–2.10)	0.188
Number of collected Oocytes	8.25 ± 5.14 (2–22)	10.50 ± 5.20 (2–23)	0.140
Metaphase II Oocytes	6.57 ± 4.22 (2–17)	7.06 ± 4.77 (2–21)	0.150
Number of day 3 embryos	3.18 ± 2.40 (2–12)	3.84 ± 2.65 (2–12)	0.120
Number of blastocyst for vitrification	0.65 ± 1.51 (0–6)	0.86 ± 1.71 (0–9)	0.200

(Positive Group, *N* = 46 and Negative Group, *N* = 123)

Descriptive statistics between two Groups. Mean values with standard deviation (SD)

Women were divided into two groups depending on the value of hCG at Day 12 after embryo transfer and ultrasound confirmation of clinical pregnancy: 123 on the negative group (72.8%) and 46 on the positive group (27.2%).

There were no statistical difference between the two set groups in terms of demographics and ART parameters: mean age of female partner or mean age of male partner, duration and type of infertility, total drug dose used for ovarian stimulation, overall median number of harvested oocytes per cycle defined as the total number of oocytes harvested during oocyte pick up procedure, rate of collected metaphase II (MII) oocytes. Also, the mean number of cleaved embryos at day 3 of embryo development, and mean number of blastocysts for cryopreservation showed no significant statistical difference between the two set groups.

Endometrial morphology showed no statistically significant difference between the two set groups.

Endometrial thickness on a single 2D sagittal profile showed no statistical difference at baseline and at Day 6 after ovarian controlled stimulation. Statistically significant difference was only met at a later phase of ovarian controlled stimulation – at day 8 and on following evaluations.

Uterine Volume was comparable between the two Groups with no statistical difference between the two. (Table 2).

**Table 2:** Ultrasound parameters between two Groups - Endometrial morphology and Endometrial thickness at baseline, at day 6, 8 and 10 after controlled ovarian stimulation, at trigger day and at embryo transfer day

		Negative Group *N* = 123 (72.8%)	Positive Group *N* = 46 (27.2%)	*p* Value
Basal	Endometrial Morphology (ML/NM)	0% / 100%	0% / 100%	NS
	Endometrial Thickness (in mm)	4.32 ± 0,72	4.22 ± 0,51	0.387
Day 6 after Controlled Ovarian Stimulation	Endometrial Morphology (ML/NM)	78.9% / 21.1%	93.5% / 6.5%	0.15
	Endometrial Thickness (in mm)	6.32 ± 0.96	6.28 ± 0.75	0.827
Day 8 after Controlled Ovarian Stimulation	Endometrial Morphology (ML/NM)	100% / 0%	100% / 0%	NS
	Endometrial Thickness (in mm)	7.47 ± 0.80	7.96 ± 0.79	**0.01**
Day 10 after Controlled Ovarian Stimulation	Endometrial Morphology (ML/NM)	100% / 0%	100% / 0%	NS
	Endometrial Thickness (in mm)	8.01 ± 1.04	8.61 ± 0.98	**0.01**
Trigger Day with rhCG	Endometrial Morphology (ML/NM)	100% / 0%	100% / 0%	NS
	Endometrial Thickness (in mm)	8.53 ± 1.32	9.59 ± 1.44	**0.001**
Embryo Transfer Day	Endometrial Morphology (ML/NM)	4.1% / 95.9%	4.3% / 95.7%	0.613
	Endometrial Thickness (in mm)	9.06 ± 1.30	10.15 ± 1.35	**0.001**

Ratios in percentages (%) and mean values with standard deviation (SD). *NM* Non multi-layered endometrium; *ML* Multi-layered endometrium; *rhCG* recombinant human chorionic gonadotropin; *NS* No statistical analysis performed

**Fig. 2:**
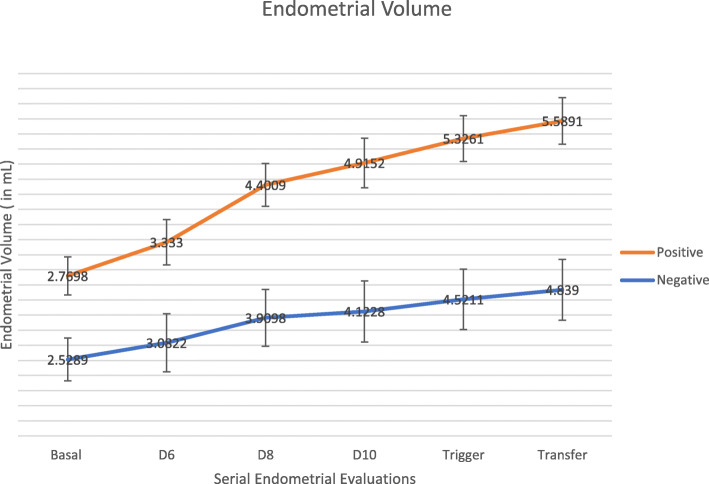
Continuous endometrial volume analysis (Mean values with Standard Deviation)

**Fig. 3:**
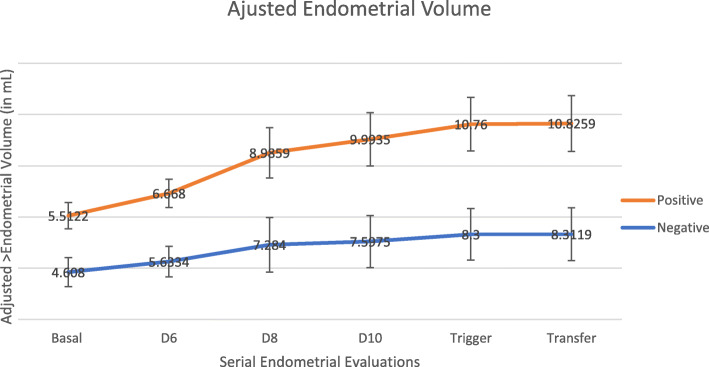
Continuous adjusted endometrial volume analysis. (Mean values with Standard Deviation)

Endometrial Volume and Adjusted Endometrial Volume showed statistical difference from Day 6 after Ovarian controlled stimulation (Table 3 and Figs. 2 and 3). Consistently higher values were seen, for both ultrasonographic markers on the positive group. In terms of endometrial volume, the positive group had statistically significant higher values in all observations except at the basal moment prior to ovarian controlled stimulation (2.77 ± 0.63 vs 2.52 ± 0.71 with *p* value of 0.54). Similar findings were noted on adjusted endometrial volume (Table 3).

**Table 3:** Ultrasound parameters between two groups - Endometrial volume and adjusted endometrial volume at baseline, at day 6, 8 and 10 after controlled ovarian stimulation, at trigger day and at embryo transfer day

		Negative Group *N* = 123 (72.8%)	Positive Group *N* = 46 (27.2%)	t-Test *p* Value
Basal	Endometrial Volume (in mm3)	2.52 ± 0.71	2.77 ± 0.63	0.54
	Adjusted Endometrial Volume	4.60 ± 1.42	5.51 ± 1.28	0.21
Day 6 after Controlled Ovarian Stimulation	Endometrial Volume (in mm3)	3.08 ± 0.66	3.33 ± 0.57	**0.024**
	Adjusted Endometrial Volume	5.63 ± 1.50	6.67 ± 1.38	**0.001**
Day 8 after Controlled Ovarian Stimulation	Endometrial Volume (in mm3)	3.90 ± 0.94	4.40 ± 0.71	**0.002**
	Adjusted Endometrial Volume	7.28 ± 2.67	8.98 ± 2.47	**0.001**
Day 10 after Controlled Ovarian Stimulation	Endometrial Volume (in mm3)	4.12 ± 1.01	4.91 ± 0.82	**0.001**
	Adjusted Endometrial Volume	7.60 ± 2.54	9.99 ± 2.61	**0.001**
Trigger Day with rhCG	Endometrial Volume (in mm3)	4.52 ± 1.00	5.33 ± 0.76	**0.001**
	Adjusted Endometrial Volume	8.30 ± 2.52	10.76 ± 2.62	**0.001**
Embryo Transfer Day	Endometrial Volume (in mm3)	4.84 ± 1.01	5.59 ± 0.77	**0.001**
	Adjusted Endometrial Volume	8.32 ± 2.58	10.83 ± 2.73	**0.001**

Ratios in percentages (%) and mean values with standard deviation (SD). *rhCG* recombinant human chorionic gonadotropin

**Fig. 4:**
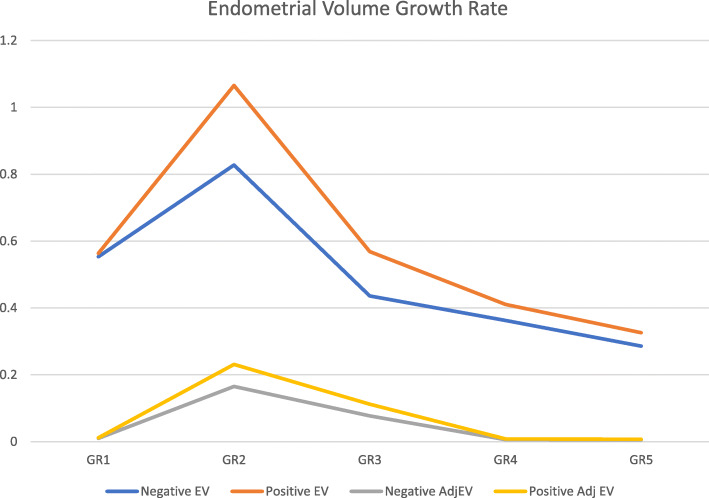
Endometrial and Adjusted Endometrial Volume Growth Rate (difference between two consecutive measurements). Negative EV – Negative Group of Endometrial Volume; Positive EV – Positive Group of Endometrial Volume; Negative AdjEV – Negative Group of Adjusted Endometrial Volume; Positive AdjEV – Positive Group of Adjusted Endometrial Volume

By comparing the difference between two consecutive measurements (endometrial growth rate) for endometrial and adjusted endometrial volumes, and the overall difference between the final value and the initial basal measurement we were able to note with statistical difference that values were higher on the positive group on initial phases of endometrial development and in the overall assessment (Table 4 and Fig. 4).

**Table 4:** Endometrial and Adjusted Endometrial volume Growth rate (difference between two consecutive continuous evaluations, and Overall difference between final and first evaluation)

Endometrial Volume Growth Rate						
	GR1	GR2	GR3	GR4	GR5	Overall GR
Negative EV	0,5533	0,8275	0,4357	0,3626	0,2861	24,654
Positive EV	0,5632	10,657	0,5686	0,4108	0,326	29,367
p Value	1,34	**0,01**	0,74	0,987	0,567	**0,01**
**Adjusted Endometrial Volume Growth Rate**						
	GR1	GR2	GR3	GR4	GR5	Overall GR
Negative AdjEV	0,0102	0,165	0,077	0,0063	0,005	0,0459
Positive Adj EV	0,0115	0,231	0,112	0,0083	0,007	0,061
p Value	1,43	**0,01**	0,53	0,873	0,678	**0,01**

*GR1* Growth rate 1 (difference between Basal Moment and Day 6 after controlled ovarian stimulation) GR2 – Growth rate 2 (difference between Day 8 and Day 6 after controlled ovarian stimulation) GR3 – Growth rate 3 (difference between Day 10 and Day 8 after controlled ovarian stimulation) GR4 – Growth rate 4 (difference between Trigger with hCG day and Day 10 after controlled ovarian stimulation) GR5 – Growth rate 5 (difference between Transfer Day and Trigger with hCG day) Overall GR – Overall Growth day (difference between embryo transfer day and Basal moment) Negative EV – Negative Group of Endometrial Volume; Positive EV – Positive Group of Endometrial Volume; *Negative AdjEV* Negative Group of Adjusted Endometrial Volume; *Positive AdjEV* Positive Group of Adjusted Endometrial Volume. Value of p < .05 considered statistically significant

In terms of endometrial volume assessment, a cut off ≥5 mL in the prediction of endometrial receptivity was used with good sensitivity (85%) and low specificity (69%) in a group application; in individual setting it had a good predictive negative value (90.1%) and low predictive positive value (81.1%), with a diagnostic accuracy of 75%.

The degree of agreement was assessed by calculation of Kappa (κ) statistics. κ is a statistical parameter of agreement that does not require any assumption of the correct diagnosis, expressed through a coefficient ranging between − 1.0 and + 1.0. Perfect agreement corresponds to a coefficient of 1.0, a value of zero indicates agreement no better than that expected by chance, and negative values indicate agreement worse than that expected by chance. There is no absolute way to interpret the values between 0 and 1. As a guideline Landis and Koch indicated that values of < 0.20 suggest poor agreement, 0.21–0.40 fair agreement, 0.41–0.60 moderate agreement, 0.61–0.80 good agreement and > 0.81 excellent agreement. In this study the intra-observer reliability was 0.96.

In addition, because all measurements were performed by the same operator in this study there was no inter-observer variability.

## Discussion

In this study we aimed to assess endometrial evolution in order to ascertain a plausible predictive non-invasive diagnostic tool for clinicians to better understand endometrium changes.

The pregnancy potential of good quality embryos is still not high on ART cycles, even with the progress in the programs of ovarian stimulation, ART technique and embryo development and culture. Implantation is still strongly reliable on the cross talk between a healthy good quality embryo and the receptive endometrium.

Although several parameters have been used to assess the pregnancy rate in ART cycles, there is still some controversy about its efficacy, and underlying mechanisms in endometrial receptivity [15–18]. Vaginal 3D ultrasound is a non-invasive and an inexpensive tool at clinician’s disposal [19]. The process of endometrial transformation from proliferative phase to secretory phase under the steroids hormonal influence, called endometrial decidualization is a set goal for optimal implantation. The cyclic changes of endometrium are regulated by ovarian hormones and its receptors, and endometrial luteal phase development may alter in ART cycles due to supraphysiological hormone levels.

Contradictory findings is often reported in single analysis of endometrial pattern at trigger day.

Recent studies (*Silva Martins, R.* et al.) have proven that perhaps serial evaluations provide better understanding rather than a single scoop at a pre-determined phase of the process. It has been proven that in terms of angiogenesis that there is a certain pattern of evolution. This relates to what one should expect from a transforming living tissue and its natural adaptations on the complex binding process of implantation.

The main purpose of this study was to further evaluate potential ultrasonographic markers that might be evaluated in the continuous changes that endometrium goes through during an ART cycle. The possibility to use non-invasive techniques to determine endometrial receptivity would allow better clinical judgment. This way on the clinical point of view, the decision to transfer a fresh embryo on that same ART cycle or postpone it for a deferred transfer with better endometrial conditions is possible. This non-invasive tool to predict endometrial receptivity will improve clinical setting and allow better understanding of endometrial receptivity. This may be the way to optimize and achieve greater results in ART cycles.

In our study endometrial morphology proven not to be useful and no significant difference was found between the two groups. Also 2-D endometrial thickness showed no difference at early stages of ovarian controlled stimulation, but significant difference could be seen after day 8 of stimulation. These findings are compatible to the ones provided by the literature. The main reason for such may be the fact that subjective tools produce conflicting results and therefore are not able to provide an accurate diagnostic tool for endometrial receptivity assessment.

Endometrial volume and adjusted endometrial volume proven to be more effective with differences shown since early stages of ovarian controlled stimulation. Both groups were similar at baseline but as soon as controlled ovarian stimulation started, the differences between the ones with a positive outcome and the negative group were clearly met.

We have also been able to show differences between the two groups in terms of endometrial and adjusted endometrial volume in early stages of endometrial development under the influence of controlled ovarian stimulation. Higher volumes were seen in the positive controls, but the changes were more evident in early stages (especially between day 6 and day 8 of ovarian controlled stimulation). This is also corroborated by the fact that growth rate was statistically higher on the positive group, but the higher difference was met in early stages of endometrial development (between day 6 and day 8). The supraphysiological environment produced by the controlled ovarian stimulation may have leading role in such. Our study showed that serial continuous endometrial volume was significantly higher on the positive group, whereas studies from Kupesic et al., and Wu et al., [20, 21] have conflicting results in the assessment of endometrial receptivity. The difference can possibly be explained by the fact of serial continuous evaluations better reflect endometrial changes, rather than predetermined single scoop analysis.

The use of a cut off ≥5 mL in endometrial volume in prediction of endometrial receptivity had a good sensitivity and low specificity and may be used as a good test to exclude success. The fact that we can have a tool that indicates a non-optimal endometrium aids in the decision process of postponing embryo transfer for a more suitable and receptive endometrium.

The possibility of real time non-invasive continuous assessment of the endometrium further induces clinicians to better medical decisions. The current study demonstrated that it is possible to evaluate endometrial morphologic parameters, serial endometrial volume and adjusted serial endometrial volume in the coronal plane in accordance with published method by Mercer et al. [22]

All findings may prove to be a useful management tool for clinicians in order to establish a diagnostic tool for better decision making in selective embryo transfers.

Nevertheless, one must always be cautious that artefacts during 3D analysis may occur due to 2D imaging process, patient motion during rendering of images and artefacts due to operator choice in the selection of which part of the volume to display [23].

We can highlight as the major strength of this study the awareness to a controversial topic, with a different uptake on the question of non-invasive methods to assess endometrial receptivity. This new methodology of continuous or better yet consecutive serial evaluations ascertain the discriminatory value of volumetric endometrial ultrasound assessment.

Also, the fact of prospective continuous assessment using the same protocol for all subjects can be pointed out as a strength of this study.

The limited number of subjects of our sample can be deemed as a limitation or weakness to this study. Still, this new methodological approach can now be used in a larger setting to provide further information and knowledge to sustain the information already seen.

This study provides a different uptake on the question of non-invasive methods to assess endometrial receptivity. We have successfully been able to obtain limited data concerning a preferential pathway on endometrial volumetry. However larger studies should be carried out to further sustain our findings.

## Conclusions

Many have been published regarding endometrial receptivity. Still many questions remain without an answer. The possibility to accurately predict implantation is one of the most challenging ones. Progress in embryo transfer and cultures, still relies on the uncertainty of embryo implantation even in the presence of what appears to be a receptive endometrium.

Some techniques have been developed but the results are still controversial, invasive and lacking reliability.

Endometrium is a living, adaptative transformative tissue. Understanding this ability to transform, and the continuity of the process may allow better knowledge of its ability to allow the implantation process of a good quality embryo.

Ultrasonographic advances in the past decades, provides a useful tool to evaluate the morphokynetics of this transformative tissue.

Information of what makes an endometrium receptive may be the key in solving these issues, providing a diagnostic tool that will enhance ART cycles and elective embryo transfers. This will result in more effective transfers and better ART outcomes. Also the possibility to determine in real time endometrial receptivity will shorten the time to birth lapse, thus improving quality of life for infertile couples.

This study showed that endometrial 3D volume analysis as well as adjusted 3D endometrium volume may identify a receptive endometrium as soon as day 6 of ovarian controlled stimulation, and also the results show a high accuracy in detecting the non-receptive endometrium (with a high Predictive Negative Value of 90.1%). In this way clinicians may be made aware of this possibility, and further enhance its procedures with better knowledge weather or not to perform embryo transfer on that given cycle.

## Data Availability

Encrypted non-disclosure data available at Open Science Framework database for peer review purpose only. Project name Physical Biomarkers in Endometrial Receptivity with access link: https://osf.io/hr25m/?view_only=8d5f6dcb8b25420bbd9188382163e7d7

## Abbreviations

ART: Assisted Reproductive Technology
CHCB: Centro Hospitalar Cova da Beira
ER: Endometrial Receptivity
ERA: Endometrial Receptivity Array
GCP: Good Clinical Practices
hCG: human chorionic gonadotropin
ICH: International Conference Harmonisation
IU: International Units
ML: Multi-layered
NM: Non Multi-layered
SD: Standard Deviation
WOI: Window of Implantation
2D: Two Dimensions
3D: Three Dimensions
